# CMAWRNet: Multiple Adverse Weather Removal via a Unified Quaternion Neural Architecture

**DOI:** 10.3390/jimaging11110382

**Published:** 2025-10-30

**Authors:** Vladimir Frants, Sos Agaian, Karen Panetta, Peter Huang

**Affiliations:** 1 Department of Electrical and Computer Engineering, Tufts University, Medford, MA 02155, USA; karen@ece.tufts.edu; 2 Department of Computer Science, College of Staten Island, City University of New York, Staten Island, NY 10314, USA; sos.agaian@csi.cuny.edu; 3 Turner–Fairbank Highway Research Center, Federal Highway Administration (FHWA), U.S. Department of Transportation, McLean, VA 22101, USA

**Keywords:** deep learning, object detection, rain removal, snow removal, quaternion image processing, quaternion neural networks

## Abstract

Images used in real-world applications such as image or video retrieval, outdoor surveillance, and autonomous driving suffer from poor weather conditions. When designing robust computer vision systems, removing adverse weather such as haze, rain, and snow is a significant problem. Recently, deep-learning methods offered a solution for a single type of degradation. Current state-of-the-art universal methods struggle with combinations of degradations, such as haze and rain streaks. Few algorithms have been developed that perform well when presented with images containing multiple adverse weather conditions. This work focuses on developing an efficient solution for multiple adverse weather removal, using a unified quaternion neural architecture called CMAWRNet. It is based on a novel texture–structure decomposition block, a novel lightweight encoder–decoder quaternion transformer architecture, and an attentive fusion block with low-light correction. We also introduce a quaternion similarity loss function to better preserve color information. The quantitative and qualitative evaluation of the current state-of-the-art benchmarking datasets and real-world images shows the performance advantages of the proposed CMAWRNet, compared to other state-of-the-art weather removal approaches dealing with multiple weather artifacts. Extensive computer simulations validate that CMAWRNet improves the performance of downstream applications, such as object detection. This is the first time the decomposition approach has been applied to the universal weather removal task.

## 1. Introduction

Intelligent transportation systems (ITS) encompass many technologies to enhance transportation safety, efficiency, and reliability [1,2]. These technologies range from advanced driver-assistance systems (ADAS) and autonomous vehicles to transport surveillance and traffic management systems.

Statistics reveal the profound impact of traffic accidents worldwide, with an alarming annual death toll of approximately 1.3 million people, and over five million injuries in the United States alone. This underscores the critical need for effective ITS solutions. Weather-related incidents, responsible for about 16% of all vehicular fatalities in the US, highlight the urgency of addressing challenges posed by adverse weather conditions like haze, rain, and snow. Images captured under these conditions are typically accompanied by low lighting conditions and adherent raindrops. To improve the situation, adverse weather image restoration has been extensively studied in the forms of dehazing [3,4,5,6,7,8,9,10], deraining [11,12,13,14], snow removal [15,16,17,18,19,20], etc. These weather conditions significantly reduce the visibility of details in images, negatively affecting the performance of computer vision algorithms, including object detection, semantic segmentation, and anomaly detection.

Prior-based methods for weather removal emerged that focus on a single weather condition [3,4,8,11,15,16]. However, it is required to address multiple types of weather simultaneously in real-world scenarios. These additional requirements complicate the design and increase the computational requirements of real-world vision systems for video surveillance and autonomous robotics. Each weather condition requires a distinct prior, based on certain assumptions, and when the assumptions are not satisfied, the performance degrades. Recently, deep-learning-based methods have become more popular due to their inherent ability to learn the priors from the data. There are trade-offs and pros and cons to using any of these approaches.

On the one hand, specialized methods demonstrate excellent performance in synthetic datasets, but the generalization ability depends on the quality and the size of the dataset, as adverse weather removal is an ill-posed problem. For many problems, such as rain streaks and snow removal, collecting large, high-quality datasets is impossible, which leads to poor performance in real-world images [21,22].

Recent methods, including MPR-Net [23], HINet [24], SwinIR [25], Uformer [26], and Restormer [27], are designed with general image restoration in mind. These methods are validated on multiple tasks, including adverse weather conditions. Nevertheless, a separately trained model is used for each condition, and removing multiple degradations is impossible with a single set of weights.

The literature on universal methods capable of removing multiple weather conditions in one step is very limited. The first work in this direction is Li et al.’s all-in-one bad weather removal network [28]. It proposes an end-to-end trained CNN with multiple convolutional encoders, one for each condition: snow, raindrops, and a combination of rain streaks and haze. Despite the ability to handle various degradations, all-in-one can only tackle one degradation at a time and has a large number of parameters, due to multiple encoders. TransWeather employs a similar technique, but uses a single visual transformer encoder and trainable weather-type query embedding to handle various degradations [29]. The network is effective in processing one weather degradation at a time. Still, performance significantly drops in the case of multiple degradations, such as severe haze and rain streaks. Chen et al. propose multiple adverse weather removal methods, trained with the help of transfer learning [30]. For each weather condition, a separate large teacher network is trained. Then, the knowledge of several teacher networks is transferred to a more compact student network. These methods indicate significant progress in universal weather removal, but are complex and computationally demanding. More information on current progress in weather removal can be found in surveys [21,22,31,32,33]. Below, we summarize the main limitations of the current state-of-the-art weather removal algorithms:

1. *Over-smoothing, unnatural color, inability to handle low-light images.* Over-smoothing commonly occurs in textured background regions due to the complex nature and high variability of degradations caused by snow, rain streaks, and raindrops. The presence of the haze generally distorts the color information and further contributes to the over-smoothing of the restored image. Although color plays an important role, deep-learning-based methods are typically trained with objective functions that either ignore color information entirely or fail to consider color space properties and inter-channel relationships. Moreover, the standard way to evaluate the methods is the application of pixel-wise metrics SSIM and PSNR on the Y channel of the YCrCb color space, which does not consider the color information [34].
2. *The limited ability to model complex patterns, such as multiple overlapping rain streaks or snowflakes and combinations of the haze and rain streaks.* The accumulation of raindrops and snowflakes at varying distances creates fog-like effects, introducing complex visual artifacts that require customized priors for effective restoration. This variability poses a significant challenge for universal weather removal models.
3. *Inability to effectively process multiple degradations.* Despite recent progress, even universal methods cannot effectively remove multiple degradations that present simultaneously in the same image.

Addressing multiple adverse weather degradations simultaneously requires a framework that can preserve the intricate relationships between color channels while processing complex, overlapping artifacts. Quaternion neural networks offer a natural solution to this challenge by representing RGB channels as a single quaternion entity, enabling the network to learn unified transformations that maintain chromatic consistency, even when haze distorts overall color balance, while rain streaks introduce localized chromatic variations. Unlike real-valued networks that process each color channel independently through separate weight matrices, QNNs employ Hamilton product operations that explicitly encode inter-channel dependencies, making them particularly effective when different weather phenomena corrupt color information in spatially and spectrally diverse ways: for instance, blue-tinted haze in background regions, combined with the wavelength-dependent scattering from water droplets in the foreground. This unified color processing, combined with the parameter efficiency of quaternion convolutions (requiring approximately four times fewer parameters than equivalent real-valued networks), makes QNNs an ideal foundation for a universal weather removal architecture that must handle diverse degradation combinations without sacrificing color fidelity or computational efficiency.

In this work, we propose CMAWRNet, a universal weather removal method that addresses visibility degradation in adverse weather and lighting conditions. CMAWRNet incorporates quaternion color representation, separate texture–structure decomposition, and attentive fusion with low-light correction. Quaternions are four-dimensional extensions of complex numbers. A quaternion has one real and three separate imaginary components, and enables the processing of color value as a single entity [35]. The quaternion color representation replaces a triplet of color channels (R, G, B) with a single quaternion number, preserving relationships between R, G, and B [28,29,35]. Quaternion neural networks take a quaternion-valued image as input and produce quaternion-valued output using the rules of quaternion algebra. Studies have shown that quaternion neural networks (QNNs) (i) are effective in cases when the real-valued neural networks (RVNNs) fail to capture the color information [36,37,38], (ii) have already shown certain advantages over RVNNs in speech, image compression, objective image quality assessment, and image classification [35,39,40,41,42,43,44], (iii) deliver a state-of-the-art performance in various tasks by reducing the number of training parameters and explicitly modeling the inter-channel correlation [45], and (iv) enable effective learning of the inter-channel and spatial relations between multiple input feature maps. So, a QNN makes a reasonable basis for a unified architecture for multiple adverse weather removal, as presented in this paper.

To effectively handle multiple adverse weather conditions, it is important to recognize how different phenomena affect specific image components. Inspired by the Retinex theory, we decompose the input image U into illumination (L) and reflectance (R) components, where U=R∘L and ∘ denotes element-wise multiplication. The illumination component captures the smooth overall scene structure, whereas the reflectance component contains the fine textures and details of objects. By processing these components separately, we can target specific degradations more effectively. Haze primarily affects the illumination component by reducing visibility and contrast across the scene. In contrast, rain streaks, raindrops, and snowflakes mainly corrupt the reflectance component by introducing a high-frequency noise that obscures textures. Leveraging local derivatives—where larger values indicate texture changes and smaller ones correspond to smooth structures—allows for accurate decomposition of the image content. This approach enables us to apply specialized enhancement techniques to each component, resulting in improved restoration quality.

This paper aims to develop a quaternion convolutional neural network called CMAWRNet for multiple degradation removal, to tackle the abovementioned challenges. Our main contributions are as follows.

1. We introduce the following:
  - *CMAWRNet*: A computationally efficient unified quaternion network architecture, designed to remove multiple degradations caused by adverse weather conditions.
  - A novel quaternion similarity loss function that preserves color information while optimizing the network.
  - DNet: A sub-network for image decomposition into texture and structure components. TNet: A lightweight transformer encoder–decoder sub-network that leverages the unique properties of quaternion neural networks. FNet: An attention-based quaternion neural network (QNN) that fuses texture and structure information, while simultaneously correcting low-light conditions.
2. We provide comprehensive experimental results, demonstrating the effectiveness of CMAWRNet in removing multiple degradations from input images. These experiments were conducted on multiple datasets and real-world images, showing superior performance in texture detail preservation and overall image quality compared to state-of-the-art methods.

## 2. Background

### 2.1. Adverse Weather Removal

Existing image restoration approaches include single weather removal algorithms for dehazing [3,4,5,6,7], deraining [8,11,12,13,14], desnowing [15,16,17,18], adherent raindrop removal [13,46], multi-degradation removal [23,47], and universal methods (all in one strategy, addressing multiple degradations at the same time) [29,30].

Single-weather-condition removal methods focus on addressing a single type of adverse weather condition, such as rain or fog. For haze removal, Cai et al. designed CNN for medium transmission map estimation and removed the haze using the atmospheric scattering model [5]. Li et al. developed a lightweight CNN for end-to-end haze removal, without explicitly estimating the transmission function [6]. Mei et al. propose a more sophisticated end-to-end dehazing architecture, employing UNet-like architecture and progressive feature fusion [48]. FFA-Net introduces an attentive feature fusion to give more weight to essential features and improve the dehazing result [49]. MSNet uses multiscale feature maps for higher spatial resolution and better contrast [50]. RefineDNet employs a two-stage strategy, using the dark channel prior for visibility restoration and weakly supervised CNN to remove artifacts introduced by the dehazing procedure and improve the realness measure [7]. For rain removal, Fu et al. introduce a dual graph convolutional network with a long-range contextual information aggregation mechanism to process long rain streaks efficiently [51]. Wang et al. narrow the domain gap between the real-world rain images and synthetic ones used for training by introducing novel physics-based rain generation procedures [52]. Qian et al. introduced a dataset for adherent raindrop removal and an attentive GAN for a single image raindrop removal [13]. Quan et al. introduce CNN with a double attention mechanism for the accurate localization of the raindrops and channel re-calibration to improve the processing of raindrops of various shapes [53]. Quan et al. propose CCN—a complementary cascaded network architecture, to remove rain streaks and raindrops in a complementary fashion via a neural architecture search [46]. For snow removal, Liu et al. proposed a synthetic dataset Snow100K, and DesnowNet—a multistage, multiscale CNN for the removal of opaque and translucent snow particles [17]. Chen et al. introduce a novel snow model, including the veiling effect and a transparency-aware convolutional architecture JSTASR [54]. DDMSNET uses semantic and geometry information in a three-stage coarse-fine snow removal framework [52]. Ye et al. developed an efficient pyramid network for real-time high-resolution image snow removal [18]. Chen et al. propose a scale-aware transformer encoder–decoder network with context interaction [19]. Despite the excellent performance of the single-weather-condition removal methods, they often include weather-specific blocks and priors and generally do not perform well on other tasks.

Multi-degradation removal offers a general image enhancement architecture that can be repurposed for any specific degradation. MPRNet uses a three-stage framework with shared features [23]. Chen et al. investigate the role of the normalization layer in the performance of multiscale, multistage architecture on low-level image processing tasks [24]. SwinIR offers a baseline transformer-based architecture instead of commonly used CNNs for image super-resolution, JPEG-compression artifact reduction, low-light image enhancement, etc. [25]. Wang et al. introduce a novel locally enhanced window transformer block and a learnable multiscale restoration modulator in an architecture called Uformer [26]. Zamir et al. introduce Restormer—an efficient and effective transformer-based architecture for low-level image processing [27]. These methods deliver a better or comparable performance to weather-specific methods, but each specific task requires a distinct set of weights and, sometimes, a specialized training procedure.

Universal (all-in-one) methods handle multiple weather conditions employing fixed architecture and weights. As a first attempt to develop a universal multi-weather removal network, Li et al. proposed the all-in-one method [28]. All-in-one takes an image degraded by any weather condition and predicts a clean image. A separate encoder, determined by a neural architecture search, is used for each weather type. TransWeather builds on the same idea, but instead of multiple convolutional encoders, a single visual transformer encoder and a decoder with weather-type embedding are applied [29]. Chen et al. introduce a novel collaborative knowledge transfer method [30]. They train a compact CNN to remove multiple weather conditions by transferring knowledge from large-scale specific-weather-type neural networks. Zhang et al. propose a universal enhancement network to improve further perception results [55]. Though these methods can achieve encouraging results in several weather types, they are ineffective in the case of a mix of different weather conditions.

### 2.2. Quaternion Neural Networks

A quaternion number $\hat{q}=a+bi+cj+dk$ extends the concept of the complex number having one real (a) and three imaginary (b, c, d) components, where a,b,c,d∈R and unity vectors i,j,k ($i^{2}=j^{2}=k^{2}=ijk=-1$) form the quaternion basis. The color input image of the size W by H pixels is represented as a quaternion matrix $\hat{I}\in H^{H\times W}$:

(1) $$\hat{I}=L+Ri+Gj+Bk$$

where $L,R,G,B\in R^{H\times W}$ are real-valued matrices representing luminosity, red, green, and blue channels. Similarly, intermediate feature maps are represented as a group of quaternion-valued matrices.

The quaternion algebra on H defines operations among quaternion numbers: addition, conjugation, and absolute value, similar to the algebra on complex numbers [35,39]. The Hamiltonian product defines the non-commutative multiplication of two quaternions $\hat{x}=a_{1}+b_{1}i+c_{1}j+d_{1}k$ and $\hat{y}=a_{2}+b_{2}i+c_{2}j+d_{2}k$ as:

(2) $$\hat{x}⊗\hat{y}=\left(a_{1}a_{2}-b_{1}b_{2}-c_{1}c_{2}-d_{1}d_{2}\right)\;\;+\left(a_{1}b_{2}+b_{1}a_{2}+c_{1}d_{2}-d_{1}c_{2}\right)i\;\;+\left(a_{1}c_{2}-b_{1}d_{2}+c_{1}a_{2}+d_{1}b_{2}\right)j\;\;+\left(a_{1}d_{2}+b_{1}c_{2}-c_{1}b_{2}+d_{1}a_{2}\right)k.$$

In QNNs, the Hamilton product replaces the real-valued dot product as the transformation between two quaternion-valued feature maps. It ensures the maintenance and exploitation of relations within components of a quaternion feature map.

The convolution of the input $\hat{q}=q_{0}+q_{1}i+q_{2}j+q_{3}k$ and kernel $\hat{W}=W_{0}+W_{1}i+W_{2}j+W_{3}k$ is defined as follows:

(3) $$\hat{q^{\prime }}=\hat{W}⊗\hat{q}$$

Typically, the quaternion convolution is implemented as a grouped real-valued convolution. A C-channel quaternion feature map is represented as a 4⋅C-channel real-valued feature map. The first C channels represent real components of quaternion feature maps, and the following three groups of C channels each represent i, j, and k-components. The components of weight $\hat{W}$ are convolved with multiple quaternion inputs.

The quaternion representation’s joint modeling of RGB channels through Hamilton product operations enables the network to maintain consistent color relationships across the restoration process, preventing the color shifts and desaturation artifacts visible in the real-valued approach. This preservation of chromatic coherence is particularly critical in adverse weather removal, where degradations often distort color information non-uniformly across different image regions.

**Figure 1:**
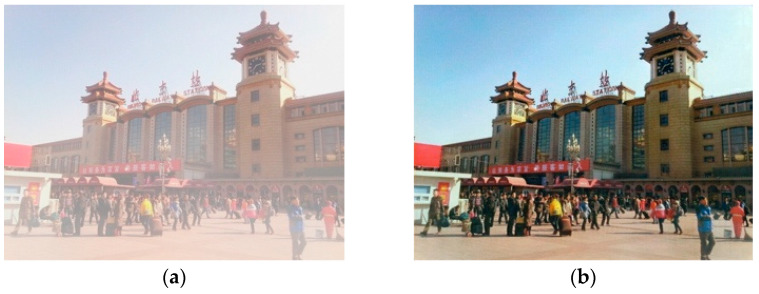

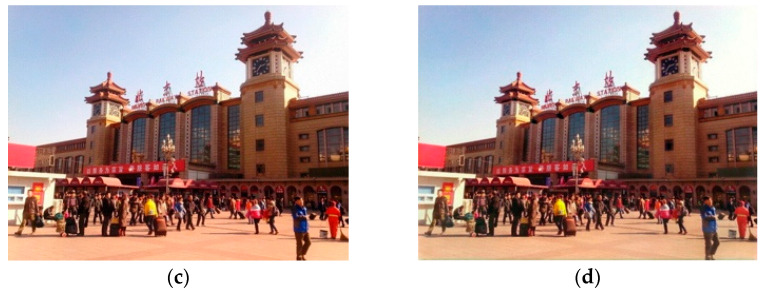
Comparison between RVNN and QNN for image restoration: (**a**) input image with degradation, (**b**) output from the RVNN (0.3 M parameters), (**c**) ground truth, and (**d**) output from the QNN (0.078 M parameters).

As can be seen from the examples in Figure 1, images produced by a quaternion neural network with 3.85x fewer parameters are almost indistinguishable from the ground truth in terms of color. This joint modeling of RGB explains the improved color constancy observed in Figure 1d versus the real-valued counterpart in Figure 1b. The QNN also preserves the structure and improves details compared to the real-valued network.

## 3. Materials and Methods

In this section, we present the CMAWRNet architecture and its key components that address critical limitations of existing universal weather removal methods. While TransWeather and Chen et al. represent significant advances in universal weather removal, they process only one weather type at a time and struggle when multiple degradations occur simultaneously, such as heavy haze combined with rain streaks, often producing images with poor color fidelity and insufficient detail recovery in low-light regions. Our approach differs fundamentally in three ways: First, CMAWRNet can handle mixed degradations, such as haze combined with rain streaks or snow with fog, in a single forward pass, whereas existing universal methods require separate processing for each degradation type. Second, we introduce structure–texture decomposition that is specifically designed for universal weather removal, enabling targeted processing of different degradation types that affect image components differently—haze primarily corrupts the structure component, while rain streaks and snow particles degrade the texture component. Third, CMAWRNet integrates quaternion-based processing throughout the pipeline with a novel QSSIM loss function to preserve color relationships, coupled with attentive fusion and gamma correction to address low-light conditions that frequently accompany adverse weather, resulting in superior color consistency compared to real-valued approaches that treat color channels independently. We begin by formulating the problem and describing the overall framework, then detail each subnetwork: DNet for structure-texture decomposition, TNet for lightweight quaternion-based transformation, and FNet for attentive fusion with low-light correction. Finally, we introduce our quaternion similarity loss function that enables better color preservation during training.

### 3.1. Problem Formulation

**Figure 2:**
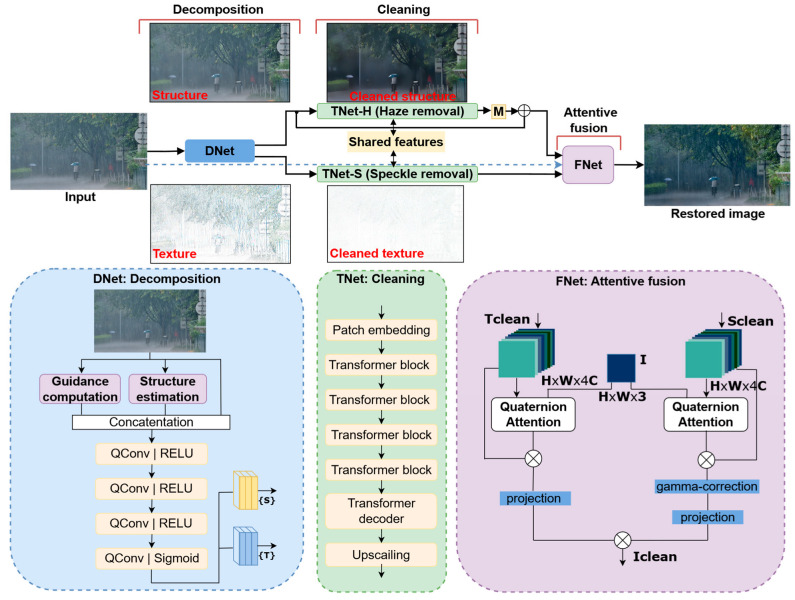
The proposed CMAWRNet framework for multiple adverse weather removal. The enhancement process is divided into decomposition, transformation, and fusion stages. In the decomposition step, DNet decomposes the input image into texture and structure components. Two separate instances of encoder–decoder TNet clean the texture and structure images with shared features to ensure adequate processing. Finally, FNet reconstructs the restored image with attentive fusion and low-light correction.

The CMAWRNet model, illustrated in Figure 2, consists of three subnetworks: DNet for image decomposition, TNet for image cleaning, and FNet for image reconstruction with gamma correction. CMAWRNet follows Algorithm 1. First, we decompose the input image I into structure S and texture T components, using the DNet subnetwork. We employ a model formulation, similar to the Retinex problem, to find an appropriate structure S by suppressing texture details T in the input image I [56]:

(4) I=S∘T

where $I\in {\left[0,1\right]}^{W\times H\times 3}$ is the original RGB image, $S\in {\left[0,1\right]}^{W\times H\times 3}$ is the structure component, $T\in {\left[0,1\right]}^{W\times H\times 3}$ is the texture component, with W and H being the width and height of the image, respectively.
**Algorithm 1** CMAWRNet Image Restoration Algorithm**Require:** Input image $I\in {\left[0,1\right]}^{W\times H\times 3}$ **Ensure:** Restored image $I_{\mathrm{clean}}$
**Initialization:** Set parameters $\gamma_{t},\gamma_{s},\gamma$, and patch size ΩCompute guidance map: $G\leftarrow {\left|\nabla I\right|}^{\gamma_{t}}$ ⊳ Equation (5)Compute structure map: $S_{0}\leftarrow \frac{1}{\left|\Omega \right|}\sum_{\left(i,j\right)\in \Omega }{\left|\nabla I\right|}_{i,j}^{\gamma_{s}}$ ⊳ Equation (6)Compute texture component: $T_{0}\leftarrow I⊘S_{0}$ ⊳ Since $I=S_{0}\circ T_{0}$**Refine components using DNet:**$S\leftarrow \mathrm{DNet}\left(S_{0}\right)$ ⊳ Refined structure$T\leftarrow \mathrm{DNet}\left(T_{0}\right)$ ⊳ Refined texture**Clean components:**$S_{\mathrm{clean}}\leftarrow \mathrm{TNet}\text{-}H\left(S\right)$ ⊳ Clean structure component $T_{\mathrm{clean}}\leftarrow \mathrm{TNet}\text{-}S\left(T\right)$ ⊳ Clean texture component**Estimate latent variable from structure:**$M\leftarrow \mathrm{Conv}1x1\left(S_{\mathrm{clean}}\right)$${\tilde{S}}_{\mathrm{clean}}\leftarrow M\circ S_{\mathrm{clean}}$${\hat{S}}_{\mathrm{clean}}\leftarrow S_{\mathrm{clean}}^{\gamma }$ ⊳ Gamma correction**Compute attention maps using FNet:**$M_{S}\leftarrow \sigma \left(W_{2}^{\left(S\right)}\mathrm{ReLU}\left(W_{1}^{\left(S\right)}\left[S_{\mathrm{clean}},I\right]\right)\right)$$M_{T}\leftarrow \sigma \left(W_{2}^{\left(T\right)}\mathrm{ReLU}\left(W_{1}^{\left(T\right)}\left[T_{\mathrm{clean}},I\right]\right)\right)$**Fuse components:**$S_{\mathrm{fused}}\leftarrow M_{S}⊗S_{\mathrm{clean}}$$T_{\mathrm{fused}}\leftarrow M_{T}⊗T_{\mathrm{clean}}$$I_{\mathrm{clean}}\leftarrow S_{\mathrm{fused}}\circ T_{\mathrm{fused}}$**return** $I_{\mathrm{clean}}$

The structure I is then processed by TNet to produce a cleaned version $S_{clean}$. Simultaneously, the texture T, representing fine surface details often degraded by rain streaks, raindrops, and snowflakes, is refined by using an encoder–decoder network to remove these artifacts, resulting in $T_{clean}$. After performing gamma correction on $S_{clean}$ to adjust illumination levels, we use attentive quaternion fusion to effectively combine $S_{clean}$ and $T_{clean}$.

### 3.2. DNet: Structure and Texture Decomposition

In this subsection, we introduce DNet, which decomposes an image I into structure S and texture T components. Following the Retinex model concept [57], we use exponentiated local derivatives to compute the guidance and structure maps. The guidance map is computed as the following:

(5) $$G={\left|\nabla I\right|}^{\gamma_{t}}$$

where $\left|\nabla I\right|$ represents the magnitude of the local gradient of the image, and $\gamma_{t}$ adjusts the influence of the gradient magnitude on G, affecting its sensitivity to texture details. We estimate the initial structure map using the local average of the exponentiated gradient magnitude:

(6) $$S_{0}=\frac{1}{\left|\Omega \right|}\sum_{\left(i,j\right)\in \Omega }{\left|\nabla I\right|}_{i,j}^{\gamma_{s}}$$

where Ω is a local patch of size 3×3 around each pixel of I, and $\gamma_{s}$ modifies the impact of the gradient magnitude in $S_{0}$, emphasizing structural components.

In our experiments, we use $\gamma_{t}=0.5$ and $\gamma_{s}=1.5$ to moderately enhance fine details without exaggerating texture components. The initial texture component is then computed as $T_{0}=I⊘S_{0}$, since $I=S_{0}\circ T_{0}$.

Finally, refining the decomposition, we apply a quaternion convolutional refinement network DNet to both $S_{0}$ and $T_{0}$. This network has three convolutional layers with ReLU activation functions, followed by a sigmoid activation function in the final layer to ensure the output range is between 0 and 1.

### 3.3. TNet: Lightweight Quaternion Encoder–Decoder

This subsection introduces TNet, a lightweight quaternion encoder–decoder network. First, we perform overlapping patch embedding on the input image of size H×W×3 [58]. Then, four quaternion transformers are used to obtain a hierarchical feature map F. We employ multi-head self-attention layers and quaternion feed-forward networks in each transformer block to calculate self-attention features:

(7) $$T_{i}\left(I_{i}\right)=\mathrm{QFFN}\left(\mathrm{QMSA}\left(Q_{i},K_{i},V_{i}\right)+I_{i}\right)$$

where $T_{i}$ represents the transformer block at stage i, QFFN denotes the quaternion feed-forward network block, QMSA stands for quaternion multi-head self-attention, and $I_{i}$ is the input at stage i in the encoder. The queries $Q_{i}$, keys $K_{i}$, and values $V_{i}$ are generated by the quaternion feed-forward network, and the attention is as follows:

(8) $$QMSA(Q_{i},K_{i},V_{i})=softmax\left(\frac{Q_{i}⊗{K_{i}}^{T}}{\sqrt{d}}\right)⊗V_{i}$$

where d represents the dimensionality, and ⊗ denotes quaternion multiplication. The computation within the quaternion feed-forward network block is summarized as the following:

(9) $${\mathrm{QFFN}}_{i}\left(X_{i}\right)=\mathrm{QMLP}(\mathrm{GELU}\left(\mathrm{DWC}\left(\mathrm{QMLP}\left(X_{i}\right)\right)\right))+X_{i}$$

where $X_{i}$ refers to the self-attention features at stage i, DWC is depthwise convolution [59], GELU is the Gaussian Error Linear Unit activation function [60], and QMLP is a quaternion multi-layer perceptron.

Features extracted by the fourth transformer for both TNet-H and TNet-S are concatenated and used as input for decoders in both instances. TNet-H and TNet-S are identical, except that TNet-H is coupled with a single ${\mathrm{Conv}}_{1\times 1}$ layer to estimate the latent variable M [6] from the equation:

(10) $$J\left(x\right)=M\left(x\right)\circ I\left(x\right)$$

where J is the restored image, I is the input image, x represents spatial coordinates, and $M\left(x\right)$ is a learnable latent variable, dependent on I [6]. The latent variable $M\left(x\right)$ is estimated as follows:

(11) $$M\left(x\right)=\frac{S\left(x\right)+tA-A}{tI\left(x\right)}$$

where A is the airlight, t is the transmission map, and $S\left(x\right)$ represents the structure component.

TNet-H generates the clean version of the structure components, while TNet-S generates the clean version of the texture components. These outputs are further fused by FNet, as shown in Figure 2.

### 3.4. FNet: Attentive Fusion

The images decomposed into structure S and texture T components need to be recombined. The cleaned features $F_{S}$ and $F_{T}$, along with the encoded features of the degraded image I, are fed into the attentive fusion subnetwork, FNet. FNet uses attention masks to produce importance weights for the fusion process. The fused feature map $F_{o}$ is computed as the following:

(12) $$F_{o}=M_{S}\circ F_{S}+M_{T}\circ F_{T}$$

where $M_{S}$ and $M_{T}$ are attention maps for the structure and texture components, respectively, and ∘ denotes element-wise multiplication.

The attention maps $M_{S}$ and $M_{T}$ for the quaternion inputs are computed as the following:

(13) $$M\left(f\right)=\mathrm{Sigmoid}\left(W_{2}\;\mathrm{ReLU}\left(W_{1}f\right)\right)$$

where $W_{1}$ and $W_{2}$ are trainable weights, and f represents the concatenated features of the input components.

The combined feature map $F_{o}$ is further fed into a ${\mathrm{Conv}}_{3\times 3}$ layer to project it to a single output quaternion image. This output is then concatenated with the input feature map and the degraded image I to produce the final restored image.

### 3.5. QSSIM Loss Function

SSIM loss was found to be used in deep learning, but SSIM ignores color information. Instead, we use the quaternion version of QSSIM, defined as the following [43]:

(14) $$QSSIM_{gt,rec}=\left(\frac{2\mu_{q_{gt}}\times \mu_{q_{rec}}}{\mu_{q_{gt}}^{2}+\mu_{q_{rec}}^{2}}\right)\left(\frac{\sigma_{q_{gt,rec}}}{\sigma_{q_{gt}}^{2}+\sigma_{q_{rec}}^{2}}\right)$$

Here “gt” means ground truth image, and “rec”—reconstructed image. Quaternion means $\mu_{q_{gt}}$, $\mu_{q_{rec}}$, variances $\sigma_{q_{gt}}$, $\sigma_{q_{rec}}$ and covariance $\sigma_{q_{gt,rec}}$ are computed as in [61].

## 4. Results

In this section, we evaluate our method on several image restoration tasks, including snow removal, rain-streak removal, and adherent raindrop removal on large, publicly available, benchmarking synthetic and real-world datasets, and compare the performance to state-of-the-art algorithms.

### 4.1. Dataset and Training

The CMAWRNet is implemented in PyTorch 1.13 [62] and trained in two steps on a single NVIDIA Tesla A100 GPU. First, the DNet and FNet are trained to perform the texture/structure split and low-light correction on the LOL (Low-Light) dataset [63]. The LOL dataset consists of 500 image pairs of low-light and normal-light scenes, primarily indoors, with a resolution of 400 × 600 pixels. It is divided into 485 training pairs and 15 testing pairs. The model is trained for 100 epochs, starting with a learning rate of 0.001, halved every 25 epochs.

In the second stage, we train the entire network using a combination of bad weather datasets. The “RainDrop” dataset contains 1119 pairs of clean images and images with adherent raindrops [13]. The “Snow100K” dataset provides 50,000 training images and 50,000 validation images with synthetic snow [17]. The “Outdoor-Rain” dataset consists of 9000 training samples and 1500 validation samples, combining synthetic rain streaks with haze. Since the datasets vary in size, we sample 9000 random images from “Snow100K” and oversample the “RainDrop” dataset by applying random data augmentation, including rotation, random cropping, and affine distortion. During the first 50 epochs, the weights of DNet and FNet are frozen. The initial learning rate is set to $1\times 10^{-3}$ and is gradually reduced to $1\times 10^{-7}$, using a cosine annealing strategy over a total of 200 epochs. During training, we randomly sample 256 × 256 pixel patches from the original-resolution images. At inference time, our model processes images of varying sizes by dividing them into overlapping blocks.

### 4.2. Comparison with State-of-the-Art

We compare CMAWRNet to state-of-the-art methods on synthetic and real-world image datasets. The quantitative results are evaluated with PSNR and SSIM [34].

**Figure 3:**
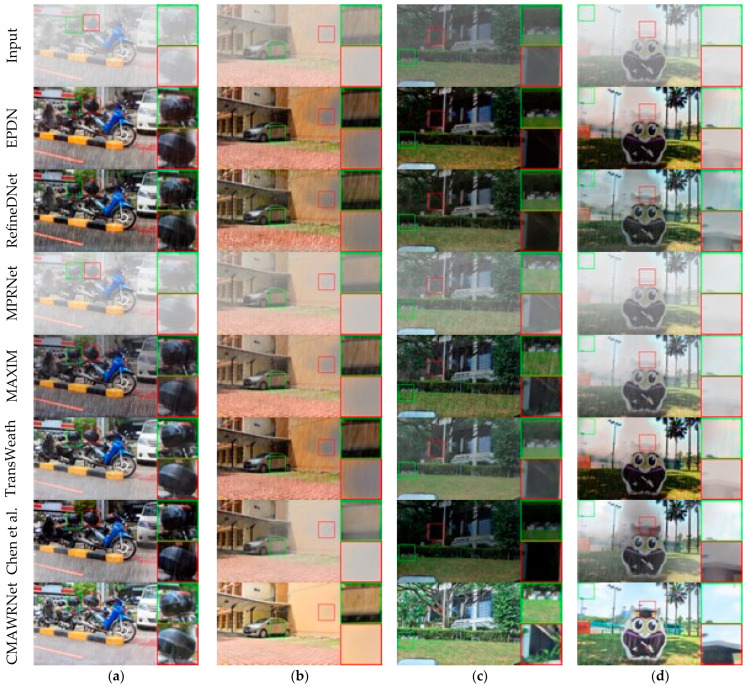
Comparison of synthetic rain and haze removal methods on the Test1 dataset: (**a**–**d**) show four test images processed by different methods (columns represent input, EPDN [64], RefineDNet [7], MPRNet [23], MAXIM [47], TransWeather [29], Chen et al. [30], and CMAWRNet). Specialized haze removal techniques (EPDN and RefineDNet) effectively remove haze, but leave rain streaks and produce darker images with loss of detail in dark regions, particularly struggling with non-homogeneous haze structures like in (**d**). MPRNet removes rain streaks, but yields low-contrast images with residual fog. MAXIM is more effective at fog removal, but struggles with rain streaks. CMAWRNet effectively reduces both fog and rain degradations, producing vivid images with preserved details, particularly in dark regions.

Rain and Fog: We evaluate the method on the synthetic dataset Test1 (part of Outdoor-Rain) [61]. We compare with baseline methods for dehazing—EPDN [64] and RefineDNet [7]; multi-degradation rain-removal—MPRNET [23] and MAXIM [47]; and universal—TransWeather [29] and Chen et al. [30]. Figure 3 shows that CMAWRNet effectively handles combined haze and rain streaks, producing vivid images with better visibility, particularly in darker regions. Other methods struggle with this combination. Although Chen et al. and TransWeather are suitable for various types of weather, they cannot process the combination of different weather types. The quantitative results are presented in Table 1. CMAWRNet outperforms other methods by a large margin.

**Figure 4:**
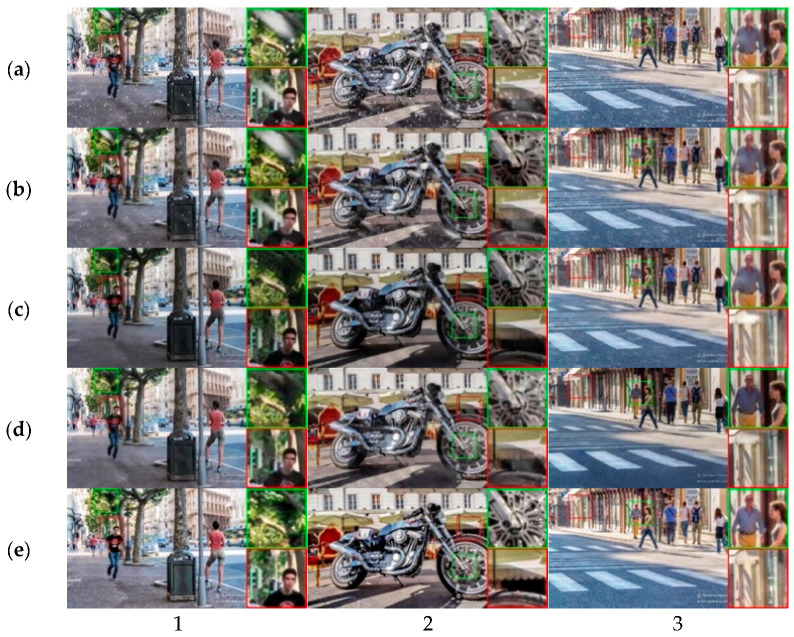
Synthetic snow removal comparison for the Snow100K dataset: (**a**) input images, (**b**) DesnowNet, (**c**) TransWeather, (**d**) CMAWRNet, (**e**) ground truth. Three test cases (1–3) are shown. In image (1), all state-of-the-art methods remove small snow particles, but DesnowNet struggles with large ones, while CMAWRNet produces realistic results. In image (2), CMAWRNet removes all snow particles and preserves small details in the wheel area better than competing methods. In image (3), only CMAWRNet successfully removes snow particles from the road, producing an image close to ground truth.

Snow: Figure 4 and Table 2 present qualitative and quantitative results for the Snow100K dataset [17]. Visual analysis demonstrates that CMAWRNet produces images with less residual haze than competing methods. Our method can remove more snow particles and rain streaks than other methods. RefineDNet effectively removes the fog but produces dark images. Also, it cannot remove snow particles in the image (1). Chen et al.’s method removes the most snow particles, but fails to remove the fog, producing dark artifacts. TransWeather removes the snow particles but does not improve visibility at all.

**Figure 5:**
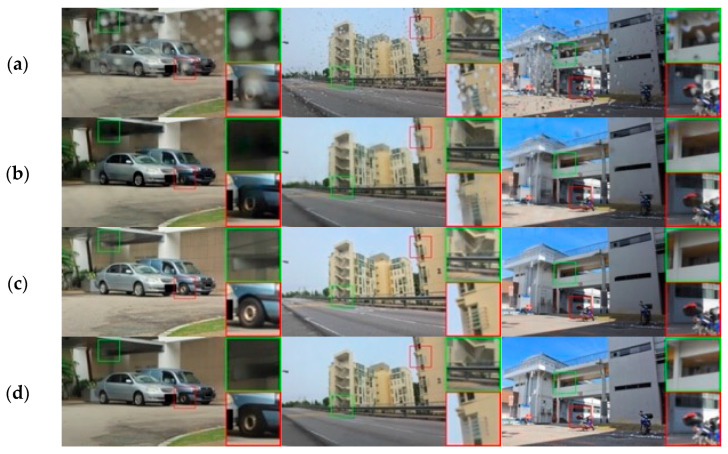
Raindrop images. (**a**) Input, (**b**)TransWeather, (**c**) CMAWRNet, (**d**) ground truth. Both TransWeather and CMAWRNet successfully remove raindrops, but CMAWRNet produces a brighter image with better details.

RainDrop Images: Figure 5 presents the simulation results of various methods tested on the RainDrop dataset [13]. The quantitative results are presented in Table 3.

Object detection: The presence of adverse weather conditions influences the functioning of downstream applications, such as object detection. To evaluate the influence of weather removal on object detection, we compare the performance of the SCNet [65] object detector on the DAWN dataset [59]. As the SCNet was trained on the COCO dataset for metrics computations, we only consider object categories from DAWN.

The DAWN dataset contains 250 real-world traffic images for each weather condition: fog, snow, and rain. Each image is annotated with bounding boxes of cars, trucks, buses, motorcycles, and pedestrians. The quantitative comparison is presented in Table 4. Mean average precision mAP and mAR are computed for the intersection of union IoU of 50% [60].

**Figure 6:**
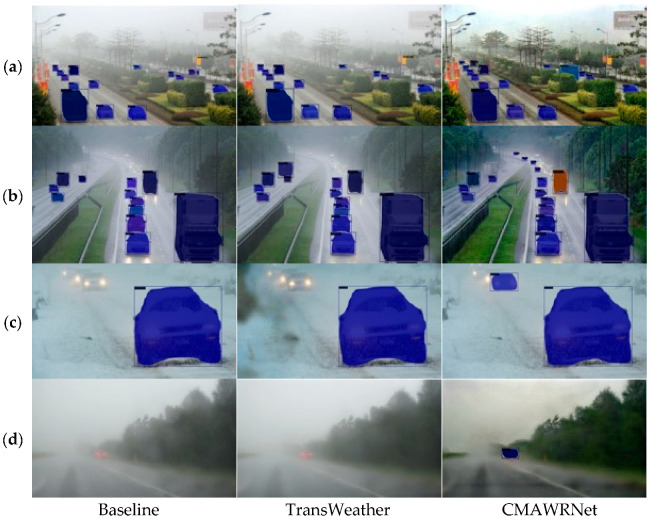
Object detection results on DAWN dataset under adverse weather conditions: (**a**–**d**) show detection in fog, rain, and snow scenarios (columns represent baseline without preprocessing, TransWeather, and CMAWRNet). CMAWRNet improves the detection of small background objects in all weather conditions, with more accurate bounding boxes and higher confidence scores compared to the baseline and TransWeather preprocessing.

As can be seen, CMAWRNet improves the detection performance in all weather conditions, especially the detection of small objects, as shown by the values of mAPs and mARs. A comparison of object detection on various real-world images is presented in Figure 6. The visual analysis shows that the result produced by CMAWRNet contains less residual haze. Also, our method can remove more snow particles and rain streaks than other methods. RefineDNet effectively removes the fog but produces dark images. Also, it cannot remove snow particles in the image (1). Chen et al.’s method removes most of the snow particles but fails to remove the fog, producing dark artifacts. TransWeather removes the snow particles but does not improve visibility at all.

**Figure 7:**
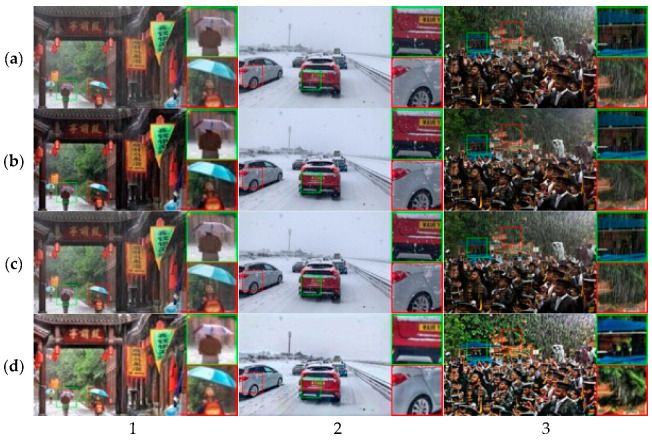
Real-world adverse weather removal comparison: (**a**) input images, (**b**) Chen et al., [30] (**c**) TransWeather [29], (**d**) CMAWRNet. Three scenarios (1–3) show haze, snow, and rain conditions. CMAWRNet generally produces vivid images with better visibility. In image (1), CMAWRNet reveals better background details. In image (2), both TransWeather and Chen et al. fail to remove snow particles, while CMAWRNet succeeds. In image (3), CMAWRNet removes more rain streaks compared to other methods.

Real Images: In Figure 7, we present the visual results recovered by the proposed method under haze, snow, and rain scenarios compared to state-of-the-art methods. Our method achieves remarkable visual quality for various types of weather.

### 4.3. Ablation Analysis

We conducted an ablation study on the “Outdoor-Rain” dataset [61] to evaluate the effectiveness of each component in our CMAWRNet architecture. Table 5 summarizes the results, including average inference times and number of parameters for different configurations, illustrating the trade-offs between accuracy and processing speed, associated with different architectural choices.

As shown in Table 5, each component of the CMAWRNet architecture contributes significantly to overall performance. The standalone transformer (TNet only) serves as a baseline, emphasizing the necessity of integrated image decomposition and feature sharing for optimal results. Excluding the image decomposition network (No DNet) demonstrates that the network relies on image decomposition for effective operation, as its absence leads to reduced performance. The configuration without shared features indicates that while individual components are beneficial, their collective operation with shared features substantially enhances results. Removing quaternion enhancements (Real-Network) highlights the integral role of quaternion algebra in managing complex image characteristics and improving quality.

Excluding the QSSIM loss function shows its importance in accurate image quality assessment and enhancement. The full architecture, which includes all components, achieves the best performance with the lowest inference time, confirming the effectiveness of integrating all developed modules.

### 4.4. Complexity Analysis

The evaluation was performed on a single NVIDIA A100 GPU. The methods compared included Chen et al. [30], TransWeather [29], and Lightweight Quaternion Chebyshev, in addition to our own.

Table 6 presents the average inference time measured in milliseconds (ms) for processing a single image of a standard resolution (1920 × 1080). CMAWRNet achieves a lower inference time than the other evaluated methods, suggesting that it is more efficient for real-time applications or scenarios, where computational resources are limited. This efficiency gain does not compromise the quality of weather removal, as demonstrated in our earlier qualitative and quantitative evaluations. By integrating advanced techniques and optimizations specific to the architecture of the NVIDIA A100 GPU, we have managed to reduce the computational burden while maintaining high performance in weather condition removal tasks.

## 5. Discussion

This work set out to answer whether a single, compact model can reliably restore images captured under multiple and mixed adverse weather phenomena, while preserving color fidelity and operating close to real time. Across three public benchmarks and a downstream perception task, CMAWRNet met those goals. The combination of (i) structure–texture decomposition (DNet), (ii) a lightweight quaternion transformer (TNet), (iii) attentive quaternion fusion with low-light correction (FNet), and (iv) a color-aware QSSIM loss produced consistent gains over the task-specific, multi-degradation, and prior “all-in-one” approaches.

**Why decomposition matters:** A key design decision was to treat adverse weather as a joint corruption, acting differently toward scene structure (e.g., global veiling from haze) and texture (e.g., high-frequency streaks and particles from rain/snow). Decomposing I=S∘T focuses TNet-H on haze/illumination and TNet-S on fine structures, before FNet fuses the cleaned components with gamma correction for visibility in dim regions. This separation proved critical in mixed weather: removing haze without washing out textures and eliminating streaks/particles without darkening the scene.

**Quantitative and visual effectiveness across conditions:** On Rain + Fog (Outdoor-Rain/Test1), CMAWRNet improved PSNR/SSIM to 30.02/0.9654, surpassing TransWeather (27.96/0.9509) and Chen et al. (28.18/0.9524); that is, a +1.84–2.06 dB PSNR and +0.013–0.015 SSIM margin for universal methods (Table 1). The qualitative rows in Figure 3 show reduced non-homogeneous haze and the removal of overlapping rain streaks, while maintaining contrast in dark regions where several baselines produced dull outputs. On Snow100K-L, CMAWRNet reached 30.08/0.9458, outpacing TransWeather (28.48/0.9308) and DesnowNet (27.17/0.8983); visually (Figure 4), it removes translucent and large opaque flakes and reduces residual veiling, even recovering road textures that others miss. On Raindrop, it achieved 32.43/0.9518, gaining +1.10–1.88 dB PSNR and +0.025 SSIM over strong task-specific and universal competitors; Figure 5 illustrates brighter, cleaner backgrounds after removing adherent drops. These results indicate that the same set of weights can generalize, from particle-like to veiling degradations, and their combinations.

**Impact on downstream perception:** Beyond restoration metrics, our pre-processing improved detection on DAWN across fog, rain, and snow. Relative to raw inputs, CMAWRNet raised mean accuracy (mA) and notably boosted small-object precision/recall (mAPs/mARs): Fog mAPs 0.132 vs. 0.076 baseline; Rain mAPs 0.198 vs. 0.115; and Snow mAPs 0.164 vs. 0.124. Figure 6 shows more small, distant vehicles correctly localized in heavy fog and precipitation, which aligns with the model’s ability to restore contrast in low-visibility backgrounds. This matters directly for ADAS/ITS pipelines that must maintain recall under adverse weather.

**Where the model helps most and where it can fail:** CMAWRNet consistently excels when haze and high-frequency artifacts co-occur, such as light fog combined with rain streaks or snow (Figure 3 and Figure 4). It also brightens dark regions without crushing blacks (Figure 3 and Figure 5), which is a frequent failure mode for dehazing-only or raindrop-only models. The remaining challenges include (i) extreme backscatter where the scene signal is faint—FNet may over-brighten, leaving slight color casts; (ii) very large, fully opaque adherent droplets or smeared lens contaminants that act as partial occluders, rather than degradations; and (iii) rare lighting (e.g., saturated highlights at night), where gamma correction alone cannot fully recover mid-tones.

**Broader implications and future work:** Universal restoration avoids the need for weather-type classification and model switching, reducing system complexity in fielded ITS/robotics stacks. Going forward, three extensions are especially promising: (1) video-aware CMAWRNet, adding temporal consistency and motion-aware fusion to stabilize sequences; (2) self-/weakly supervised training, leveraging unpaired real weather videos and physics-guided priors (airlight/transmission) to close the synthetic-to-real gap; and (3) task-aware co-training, jointly optimizing restoration with detectors/segmenters to maximize end-to-end perception gains. On the systems side, pruning/quantization for edge GPUs, and learning severity-adaptive fusion policies could further reduce latency and energy while preserving quality. Finally, extending the decomposition idea to other domains (sand/dust storms, underwater scattering) may yield a single, more general adverse-media restoration backbone.

## 6. Conclusions

To this end, we propose CMAWRNet, an efficient multistage architecture for adverse weather removal. CMAWRNet employs a unified framework that handles multiple weather conditions by decomposing input images into texture and structure components, which are processed separately and then fused. We also propose DNet—a quaternion network—for image decomposition, and FNet—a quaternion attention-based network—for low-light correction. CMAWRNet delivers significant performance gains on various benchmark datasets. We also obtain better visual results on real-world images of snow and rain.

## Figures and Tables

**Table 1:** Quantitative comparison on the test1 (rain + fog) dataset based on PSNR and SSIM.

Type	Method	PSNR	SSIM
Task-specific	EPDN [64]	13.36 ± 2.84	0.5830 ± 0.0912
	RefineDNet [7]	15.68 ± 3.12	0.6400 ± 0.0856
Multi-degradation	MPRNet [23]	21.90 ± 4.26	0.8456 ± 0.0634
	MAXIM [47]	26.91 ± 3.58	0.9212 ± 0.0428
Universal	TransWeather [29]	27.96 ± 3.42	0.9509 ± 0.0318
	Chen et al. [30]	28.18 ± 3.38	0.9524 ± 0.0305
	CMAWRNet (Our)	30.02 ± 3.15	0.9654 ± 0.0268

**Table 2:** Quantitative comparison of the SnowTest100k-L test dataset, based on PSNR and SSIM.

Type	Method	PSNR	SSIM
Task-specific	JSTASR [54]	25.32 ± 3.92	0.8076 ± 0.0845
	DesnowNet [17]	27.17 ± 3.64	0.8983 ± 0.0612
Universal	Chen et al. [30]	28.33 ± 3.48	0.8820 ± 0.0687
	TransWeather [29]	28.48 ± 3.41	0.9308 ± 0.0456
	CMAWRNet (Our)	30.08 ± 3.22	0.9458 ± 0.0398

**Table 3:** Quantitative comparison of the RainDrop test dataset, based on PSNR and SSIM.

Type	Method	PSNR	SSIM
Task-specific	Attn. GAN [13]	30.55 ± 2.18	0.9023 ± 0.0534
	Quan et al. [46]	31.33 ± 2.08	0.9263 ± 0.0445
Universal	Chen et al. [30]	28.84 ± 2.45	0.9460 ± 0.0312
	TransWeather [29]	31.12 ± 2.12	0.9268 ± 0.0438
	CMAWRNet (Our)	32.43 ± 1.95	0.9518 ± 0.0285

**Table 4:** Average mean precision and recall for subsets of DAWN dataset.

	DAWN Fog				DAWN Rain				DAWN Snow			
	mA	mAP_S_	mAR	mAR_S_	mAP	mAP_S_	mAR	mAR_S_	mAP	mAP_S_	mAR	mAR_S_
Baseline	0.548	0.076	0.422	0.183	0.520	0.115	0.467	0.221	0.593	0.124	0.436	0.212
EPDN [64]	0.551	0.082	0.426	0.187	0.523	0.118	0.472	0.228	0.598	0.127	0.442	0.216
RefineDNet [7]	0.549	0.076	0.423	0.183	0.520	0.115	0.468	0.222	0.593	0.124	0.436	0.212
MPRNet [23]	0.539	0.054	0.405	0.168	0.522	0.116	0.471	0.224	0.583	0.117	0.421	0.196
MAXIM [47]	0.550	0.080	0.427	0.189	0.518	0.113	0.464	0.216	0.594	0.127	0.424	0.215
TransWeather [29]	0.552	0.081	0.441	0.181	0.523	0.168	0.474	0.236	0.572	0.109	0.432	0.198
Chen et al. [30]	0.488	0.026	0.347	0.058	0.491	0.039	0.406	0.232	0.572	0.109	0.432	0.198
JSTASR [54]	0.547	0.076	0.422	0.183	0.520	0.115	0.467	0.221	0.598	0.128	0.439	0.217
DesnowNet [17]	0.548	0.075	0.421	0.179	0.520	0.115	0.467	0.220	0.597	0.125	0.438	0.216
Attn. GAN [13]	0.548	0.076	0.422	0.183	0.520	0.115	0.467	0.221	0.593	0.124	0.436	0.212
Quan et al. [46]	0.548	0.076	0.422	0.183	0.520	0.115	0.467	0.221	0.593	0.124	0.436	0.212
QSAM-Net [66]	0.549	0.076	0.431	0.184	0.531	0.114	0.475	0.225	0.593	0.124	0.436	0.212
LQC [67]	0.578	0.090	0.451	0.194	0.531	0.181	0.476	0.234	0.598	0.155	0.445	0.215
CMAWRNet	0.587	0.132	0.471	0.221	0.536	0.198	0.482	0.249	0.614	0.164	0.451	0.218

**Table 5:** Ablation study on Outdoor-Rain dataset, showing PSNR, SSIM, parameters, and inference time.

Configuration	DNet	Shared Features	Quaternion Enhancement	QSSIM Loss Function	Params (M)	PSNR	SSIM	Inference Time (ms)
Standalone Transformer (TNet only)	✗	✗	✗	✗	5.0	27.10	0.9320	30.5
No Image Decomposition	✗	✓	✓	✓	13.5	28.30	0.9440	28.7
No Shared Features	✓	✗	✓	✓	14.8	29.05	0.9532	26.8
Real-Network	✓	✓	✗	✓	16.2	29.62	0.9519	25.6
No QSSIM Loss Function	✓	✓	✓	✗	18.0	29.85	0.9580	24.7
CMAWRNet	✓	✓	✓	✓	18.0	30.02	0.9654	24.3

**Table 6:** Complexity analysis.

Methods	Average Inference Time (ms)
Chen et al. [30]	45.2
TransWeather [29]	37.8
Lightweight Quaternion Chebyshev [67]	29.5
CMAWRNet (Ours)	24.3

## Data Availability

The data presented in this study are available on request from the corresponding author, the data are not publicly available due to privacy restrictions.

## Abbreviations

The following abbreviations are used in this manuscript:

ADAS	Advanced Driver Assistance Systems
CMAWRNet	Quaternion-Based Universal Multi-Weather Restoration Network
CNN	Convolutional Neural Network
COCO	Common Objects in Context (dataset)
DAWN	Vehicle Detection in Adverse Weather Nature (dataset)
DNet	Decomposition Network (structure/texture split)
DWC	Depthwise Convolution
EPDN	Enhanced Pix2pix Dehazing Network
FNet	(Attentive) Fusion Network
GAN	Generative Adversarial Network
GELU	Gaussian Error Linear Unit
GPU	Graphics Processing Unit
HINet	Half Instance Normalization Network
ITS	Intelligent Transportation Systems
IoU	Intersection over Union
JSTASR	Joint Size and Transparency Aware Snow Removal
LOL	Low-Light (dataset)
LQC	Lightweight Quaternion Chebyshev (baseline)
MAXIM	Multi-Axis MLP for Image Processing
MLP	Multi-Layer Perceptron
MPRNet	Multi-Stage Progressive Image Restoration
PSNR	Peak Signal to Noise Ratio
QFFN	Quaternion Feed-Forward Network
QMLP	Quaternion Multi-Layer Perceptron
QMSA	Quaternion Multi-Head Self Attention
QNN	Quaternion Neural Network
QSAM-Net	Quaternion Self Attention Module Network (rain-streak removal)
QSSIM	Quaternion Structural Similarity Index
RGB	Red Green Blue Color Space
RVNN	Real Valued Neural Network
ReLU	Rectified Linear Unit
SCNet	Sample Consistency Network (for instance segmentation)
SSIM	Structural Similarity Index
SwinIR	Swin Transformer for Image Restoration
TNet	Lightweight Quaternion Encoder–Decoder (texture/structure cleaning)
TransWeather	Transformer-Based Adverse-Weather Restoration Model
UNet	U-shaped Convolutional Network
Uformer	U-shaped Transformer for Image Restoration
YCrCb	Luma Chroma Color Space (Y, Cr, Cb)
mAP	Mean Average Precision
mAPs	Mean Average Precision (small objects)
mAR	Mean Average Recall
mARs	Mean Average Recall (small objects)
